# Phase Locking Induces Scale-Free Topologies in Networks of Coupled Oscillators

**DOI:** 10.1371/journal.pone.0002644

**Published:** 2008-07-09

**Authors:** Irene Sendiña-Nadal, Javier M. Buldú, Inmaculada Leyva, Stefano Boccaletti

**Affiliations:** 1 Departamento de Física, Universidad Rey Juan Carlos, Móstoles, Madrid, Spain; 2 Embassy of Italy in Tel Aviv, Tel Aviv, Israel; 3 CNR- Istituto dei Sistemi Complessi, Sesto Fiorentino (Fi), Italy; Tel Aviv University, Israel

## Abstract

An initial unsynchronized ensemble of networking phase oscillators is further subjected to a growing process where a set of forcing oscillators, each one of them following the dynamics of a frequency pacemaker, are added to the pristine graph. Linking rules based on dynamical criteria are followed in the attachment process to force phase locking of the network with the external pacemaker. We show that the eventual locking occurs in correspondence to the arousal of a scale-free degree distribution in the original graph.

## Introduction

Many biological, neural, chemical and technological systems find a suitable representation as growing networks of interconnected dynamical units [1]–[3]. It is nowadays established that such networks, as they are observed in their mature state, are characterized by specific topological features, as, for instance, relatively small characteristic distances between any two nodes, high clustering properties, and fat tailed shapes in the distribution of their connectivities. In particular, most of them are known to exhibit the so-called scale-free (SF) property [4] consisting in the fact that they feature a power-law distribution *P*(*k*)∼*k* ^−*γ*^ in the node connectivity *k* (degree). From the other side, it is observed that their complex functioning and organization is often associated with the adjustment of all (or a relevant portion of) their dynamical components into a collective synchronized motion.

It is therefore crucial to understand the intimate relationship between the topological structure displayed in the resulting graphs, and the mechanisms leading to the arousal of such synchronized behaviors. For instance, recent studies have shown that *i)* the ability of a graph to give rise to a synchronous behavior can be greatly enhanced by exploiting the topological structure emerging from independent statistically driven growth processes [5]–[7]; *ii)* proper topological mechanisms of rewiring/decoupling can enhance the arousal of a synchronized behavior [8]; *iii)* a dynamical evolution of the underlying topology of a graph is eventually able to stabilize a synchronous motion also in those cases in which synchronization would be prevented in static graph configurations [9]–[11].

However, the question of how node dynamics can shape the network has not been extensively addressed. The issue has only been considered within the game theory framework [12], [13], where a not growing network of players is shaped in a decision game. In this Letter, we show that a growing process entirely guided by dynamical criteria to force frequency and phase locking of an original set of networking oscillators is able to fully reshape the connectivity of the graph, and that the entrainment process induces a scale-free degree distribution in the pristine network.

## Methods

Without lack of generality, we exemplify our discussion with reference to an initial *t* = 0 graph G_0_ of bi-directionally coupled Kuramoto phase oscillators [14], [15], ruled by 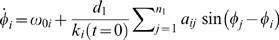 where *i* runs from 1 to *n*_1_, {*ω*_0*i*_} is the set of natural frequencies of the phase oscillators, uniformly distributed within the range 0.5±0.25, *k_i_*(*t* = 0) is the initial degree of (the number of connections pertinent to) the *i^th^* oscillator, *d*_1_ is a coupling constant, and the set {*a_ij_*} are the elements of the *n*_1_×*n*_1_ adjacency matrix **A** = (*a_ij_*) describing the structure and topology of the wiring of connections in G_0_ (*a_ij_* = 1 if oscillators *i* and *j* are connected, while *a_ij_* = 0 otherwise). Time integration is here performed by means of the Heun method with an integration step Δ*t_in_* = 0.1.

Though the main results of our study are independent on the initial choice of G_0_, from the time being we generate G_0_ by means of the model introduced in Ref. [16]. Precisely, we start with a ring lattice of *n*_1_ nodes, each one connected to its *k*_0*i*_ = 2*m*_0_ nearest neighbors, and obtain G_0_ by randomly rewiring each link with probability *p* = log *n*_1_/*n*_1_. The resulting G_0_ approximates the so called *small world* property, featuring an average shortest path length ∝ log *n*_1_, and an exponentially decaying degree distribution *P*_0_(*k*) that is well peaked around the mean value 〈*k*〉 = 2*m*_0_. Furthermore, the coupling constant *d*_1_ is selected so as the initial graph does not display a phase synchronized motion.

In our generic trials, the initial network G_0_ is let evolve in its unsynchronized motion from *t* = 0 to *t* = 30 time units, and, at subsequent times *t_l_* = *t*_0_+*l*Δ*t*, a forcing network is grown on top of the evolution of G_0_. Namely, at each time interval Δ*t* (which will be taken as a multiple of the integration step Δ*t_in_*, i.e. Δ*t* = *s*Δ*t_in_*), a new node is added, forming *m* connections with nodes in G_0_. The added nodes are identical phase oscillators that follow the instantaneous phase of an external pacemaker 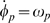, and all newly formed links correspond to unidirectional (forcing) interactions to nodes in G_0_. Notice that such forcing process is fully equivalent to considering a unique external forcing node (the pacemaker) which forms successive (and possibly multiple) connections with oscillators in G_0_ in the attempt of locking their frequencies, and, as so, it can be taken as a representation of phenomena occurring in social science [the emergence of consensus driven by a leader opinion (media, press, fashion, publicity, etc..)] or in biological systems (the entrainment of circadian rhythms driven by the main circadian clock).

The key point is the selection mechanism through which the added nodes are linked to G_0_. We here consider a dynamical criterion fully driven to enhance phase locking: when the *l^th^* new node is attached to G_0_, it forms *m* connections *preferentially* with those nodes in G_0_ whose instantaneous phase at time *t_l_*, *φ_j_*(*t_l_*), holds more closely a given phase condition. Specifically, we consider a generic parameter *δ*∈(0,2*π*) and establish the first of the *m* connections with that node *j_l_* whose actual value of the phase holds the condition 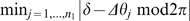 with Δ*θ_j_* = *φ_j_*(*t_l_*)−*φ_p_*(*t_l_*). When *m*>1, we iteratively repeat the same condition excluding those nodes that already received a link at the same time step. In the following, we will set *m* = 1. However, the reported scenario is independent on the specific choice of *m*, the only difference being that for *m* = 1, the added nodes do not form additional cycles nor loops in the original graph G_0_. As for the parameter *δ*, we will show that it will not affect qualitatively the reported scenario. The only constrain is that it cannot be taken equal to 0 nor to 2*π*, as these values correspond to the stable fixed point emerging during the locking of a single phase oscillator, and therefore these settings would determine a situation in which the first node in G_0_ becoming locked with the pacemaker, would, from there on, attract all the rest of connections.

Each newly formed connection is assigned a coupling coefficient *d_p_* and a coupling direction from the added node to the selected node in G_0_ (different kinds of coupling interactions have been also studied, and a detailed report will be presented elsewhere [17]). The evolution of the graph is therefore described by
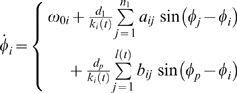
where *k_i_*(*t*) is the time evolving degree of the *i^th^* node that accounts for new connections the *i^th^* node is receiving from added nodes, and the matrix **B** = (*b_ij_*) is a size evolving matrix of *n*_1_×*l*(*t*) elements (with *l*(*t*)≤*n*_2_), whose entries *b_ij_* are equal to 1 if the *j^th^* added node formed a connection with the *i^th^* node in G_0_, and zero otherwise.

## Results

**Figure 1:**
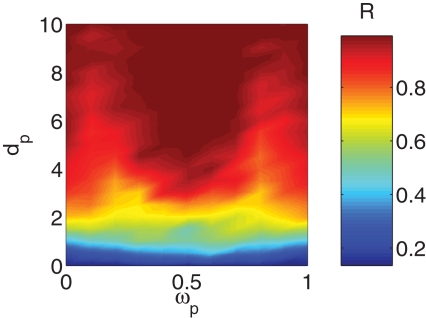
Time averaged phase synchronization order parameter *R* in the parameter space *ω_p_*−*d_p_*. Time averaged phase synchronization order parameter *R* (see text for definition) as a function of both the pacemaker frequency *ω_p_* and the coupling strength *d_p_*. Parameters: *n*_1_ = 100, *d*_1_ = 0.2, *m*_0_ = 2, *n*_2_ = 200, *δ* = *π* and each point is an average over 10 different realizations of the growing process.

Figure 1 reports the quantity *R* = 〈*R*(*t*)〉*_t_ vs*. the parameter space (*ω_p_*, *d_p_*) for *n*_1_ = 100, *d*_1_ = 0.2, *n*_2_ = 200, *s* = 1, and *δ* = *π* (anti-phase coupling condition). Here 〈…〉*_t_* denotes an average over time (performed after the growing process is finished), and *R*(*t*) stands for the phase synchronization order parameter [14] 
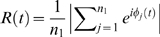
.

**Figure 2:**
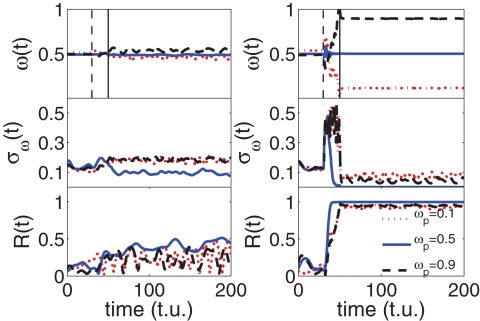
Time evolution of the entrainment process. Time evolution of the mean frequency (upper row), frequency dispersion (middle row) and phase synchronization order parameter (lower row) for three pacemaker frequencies (*ω_p_* = 0.1 red-dotted line, *ω_p_* = 0.5 blue-solid line, and *ω_p_* = 0.9 black-dashed line) and coupling strengths *d_p_* = 0.5 (left column) and *d_p_* = 5.5 (right column). See text for the definition of all reported quantities. The two vertical lines in the upper row denote the instants at which the growth process starts (dashed) and ends (continuous). Same parameters as in the caption of Figure 1.

From Figure 1 it is evident that the threshold for the setting of the phase locking of *R*(*t*) (*R*≃1) crucially depends on the frequency of the external pacemaker *ω_p_*. Specifically, as far as *ω_p_* is close to *ω̅* = 0.5 (the average frequency of the oscillators in G_0_), the locking process occurs already for a relatively small value of *d_p_*, whereas, as *ω_p_* deviates significantly from *ω̅* the value of *d_p_* producing phase locking becomes larger and larger. A more quantitative description of the process can be gathered by inspection of Figure 2, where we report the time evolution of the mean frequency of the oscillators in G_0_, 
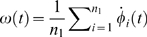
, of its frequency dispersion 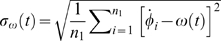, and of *R*(*t*), for three pacemaker frequencies (*ω_p_* = 0.1, 0.5 and 0.9) and two distinct coupling strengths (*d_p_* = 0.5 and *d_p_* = 5.5). It is seen that, while the low coupling regime is not associated to a phase locking of G_0_ with the pacemaker, in the high coupling regime *ω*(*t*) converges (after the growing process has ended) to the external forcing frequency and, at the same time *R*(*t*) converges to unity, and *σ_ω_*(*t*) vanishes.

The results reported in Figures 1 and 2 are generic, and the same qualitative scenario (though for different values of *d_p_* and *ω_p_*) characterizes the evolution of the system at different system sizes, for different initial topologies in the connectivity of G_0_, for different values of *δ*∈(0,2*π*) and for different values of *m*.

**Figure 3:**
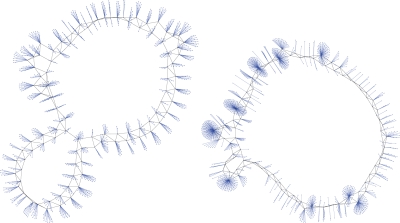
Sketches of two typical output networks. These two networks are constructed for *n*_1_ = 100, *n*_2_ = 1000, and *δ* = *π*. The nodes of the original graph are depicted in black, the forcing nodes are depicted in blue. The left (right) network corresponds to a case in which the forcing nodes are unable (able) to lock the phases of the oscillators in G_0_.

Let us now move to report the central point of this study, related to the investigation of the peculiar topological structures induced in the final degree distribution of G_0_ as a result of the phase locking process. For instance, in Figure 3 we depict the final graphs obtained for *n*_1_ = 100 and *n*_2_ = 1000, with the nodes of the original graph (the added nodes) depicted in black (blue). The left and right networks correspond to very different situations. In the left network, the growing process is unable to produce locking of the phases of the oscillators in G_0_ to the external pacemaker, and one sees that the distribution of blue attachments is rather homogeneous. At variance, the right graph corresponds to a case in which the forcing nodes eventually succeed to entrain the phases of the oscillators in G_0_, and eye inspection reveals a high heterogeneity of the blue attachments, with the simultaneous presence of few hubs (nodes with very high degree) and many seldomly connected nodes.

In order to describe more quantitatively the situation, we perform large trials of numerical simulations with *n*_1_ = 1000, *n*_2_ = 10000, and *d*_1_ = 0.2, and we monitor the time evolution of the degree distribution *P_t_*(*k*) of all nodes originally belonging to G_0_ during the process of locking. In fact, we here measure the *cumulative* degree distribution 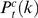, given by 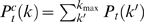. This is because the summing process of the *P*(*k*) smooths the statistical fluctuations generally present in the tails of the distribution. As a generic property, it is important to remark that, if a power law scaling is observed in the behavior of *P^c^*(*k*) (i.e. if 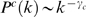), this implies that also the degree distribution *P*(*k*) is characterized by a power law scaling *P*(*k*)∼*k* ^−*γ*^, with *γ* = 1+*γ_c_*.

**Figure 4:**
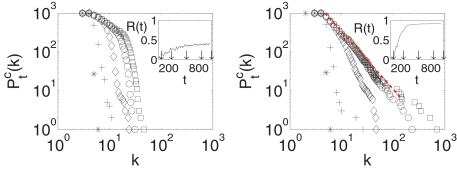
Time evolution of the cumulative degree distribution for a particular realization of the network growth. *ω_p_* = 0.5 and *d_p_* = 0.2 (left panel, non locked graph) and *d_p_* = 0.5 (right panel locked graph). In both cases the inset reports the corresponding time evolution of *R*(*t*) (see text for definition). The time instants at which the distributions are taken (indicated by arrows in the insets) are: *t* = 0 (*); *t* = 200 (+); *t* = 500 (◊); *t* = 800 (○); *t* = 1000 (□). Notice that, in the locked case, 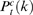 converges to an asymptotic distribution 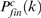 which features a power-law shape (best fit sketched with red dashed line).

Figure 4 reports how 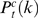 evolves in two distinct situations: for a process that does not lead to any locking (left panel) and for a process that eventually leads to locking of G_0_ to the frequency of the pacemaker (right panel). In the former case, while 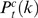 deviates significantly from 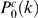 in the course of time, it never assumes a power-law shape, while in the latter case the locking process (manifested by the evolution of *R*(*t*) to 1 in the inset) is accompanied by the convergence of 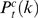 to an asymptotic distribution 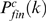 which features a power-law shape.

**Figure 5:**
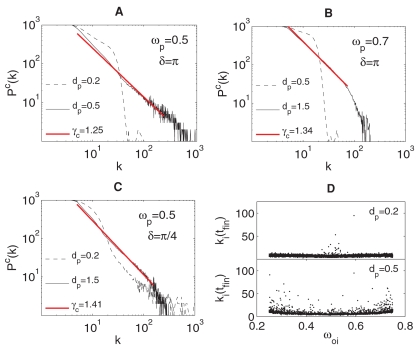
Ensemble average of the final degree distribution in the parameter space. (a)–(c): Log-log plots of 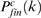 (see text for definition) *vs. k.* obtained after an ensemble average over 50 different realizations of the growing process (*n*_1_ = 1000, *n*_2_ = 10000, *d*_1_ = 0.2). In all cases, solid (dashed) lines correspond to the locked (non locked) regime, obtained for high (low) values of *d_p_*, and solid red lines indicate the best power-law fits. (d): final number of connections *k_i_*(*t_fin_*) acquired by each node as a function of its initial frequency *ω*_0*i*_ = 0.5 for, *δ* = *π*, and *d_p_* = 0.2 (upper plot, unlocked case) and *d_p_* = 0.5 (lower plot, locked case).

The difference in the final distributions for the non locked and locked networks, and the convergence in this latter case of 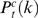 to a SF distribution 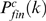 is a generic feature in the parameter space, as it can be seen in Figures 5(a)–(c). There, we report log-log plots of 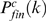 obtained after an ensemble average over 50 different realizations of the growing process for *n*_1_ = 1000, *n*_2_ = 10000, *d*_1_ = 0.2 and two distinct values of *δ* [*δ* = *π* in Figures 5(a)–(b), and *δ* = *π*/4 in Figure **5**(c)]. In all cases, solid (dashed) lines correspond to the locked (non locked) regime, obtained for high (low) values of *d_p_*, and solid red lines indicate the best power-law fits. Whenever the forcing nodes eventually induce the locking of G_0_ to the pacemaker frequency, the final degree distribution displays a power-law (scale-free) behavior *P*(*k*)∼*k* ^−*γ*^. The specific slope *γ* of the power law scaling depends on the specific choice of the external frequency *ω_p_*. In our trials, we always observed values of *γ* in the range (2,3), in accordance to the values measured for most of the real world networks [1]–[3].

## Discussion

The formation of a scale free degree distribution can be qualitatively understood as follows. Each node in G_0_ can be in two different states: a state in which its phase is locked with that of the pacemaker (corresponding to Δ*θ* = 0) and an unlocked state, in which Δ*θ*(*t*) rotates in the unit circle clockwise or counterclockwise, depending on whether the difference between the frequency of the node and that of the pacemaker is positive or negative. Having set *δ* strictly different from 0, this implies that, once a node reaches the locked state, it cannot be further attached by the pacemaker. As a given node is connected to the pacemaker, it is reasonable to assume that its phase dynamics will be slightly attracted to that of the pacemaker. Initially, all nodes are not locked, and therefore the probability to find their phase differences in the proximity of the attachment condition will not depend on their specific initial frequency. When *d_p_* is small enough so as not to determine locking of the network, only the very few nodes whose original frequency was close to that of the pacemaker will reach the locked state, whereas all the remaining nodes will evolve unsynchronized, and for them we will have a sort of random attachment with equal probability leading to a final degree distribution which substantially differs from a scale free network (Figures 5(a)–(c), dashed lines). On the contrary, at high values of *d_p_*, nodes will be sequentially attracted to the locked state, starting again from those whose original frequency was close to that of the pacemaker. In this latter situation, the more nodes gets the locked state (where any further attachment is prevented), the higher is the probability that the remaining unlocked nodes will get subsequent connections, resulting in a kind of preferential attachment process in which the higher is the absolute value of the difference between the original frequency of a node and that of the pacemaker, the higher is the chance that this node will get connections during the growing process. This is confirmed in Fig. 5(d), where we report the final number of connections *k_i_*(*t_fin_*) acquired by each node as a function of its initial frequency *ω*_0*i*_ for *ω_p_* = 0.5 and two values of *d_p_*. There, one clearly see that, while in the unlocked regime (upper plot) the distribution is almost uniform reflecting a random-like attachment, in the locked regime (lower plot), the distribution is strongly nonuniform, and promotes those nodes with higher frequency mismatch with the pacemaker. Notice that the few points in Figure 5(d) of high degree in proximity to *ω_p_* correspond to oscillators whose initial frequency was, indeed, close to *ω_p_*, but whose instantaneous frequency at the instant at which the growing process starts was moved away from *ω_p_* during the initial, unforced, evolution of G_0_.

In conclusion, we have shown that a growing process entirely guided by dynamical criteria to promote and phase locking of an original network is able in a robust way to fully reshape the connectivity of the graph, and that the locking process is associated with the emergence of a scale-free degree distribution in the network connectivity. This fact can therefore give new hints on the fundamental processes that rule the growth of some of the real world networks, that ubiquitously feature such kind of connectivity distributions.
